# The impact of malaria coinfection on Ebola virus disease outcomes: A systematic review and meta-analysis

**DOI:** 10.1371/journal.pone.0251101

**Published:** 2021-05-24

**Authors:** Hannah M. Edwards, Helen Counihan, Craig Bonnington, Jane Achan, Prudence Hamade, James K. Tibenderana

**Affiliations:** Malaria Consortium, London, United Kingdom; National Institute for Infectious Diseases IRCCS Lazzaro Spallanzani, ITALY

## Abstract

**Introduction:**

Viral outbreaks present a particular challenge in countries in Africa where there is already a high incidence of other infectious diseases, including malaria which can alter immune responses to secondary infection. Ebola virus disease (EVD) is one such problem; understanding how *Plasmodium spp*. and Ebolavirus (EBOV) interact is important for future outbreaks.

**Methods:**

We conducted a systematic review in PubMed and Web of Science to find peer-reviewed papers with primary data literature to determine 1) prevalence of EBOV/*Plasmodium spp*. coinfection, 2) effect of EBOV/*Plasmodium spp*. coinfection on EVD pathology and the immune response, 3) impact of EBOV/*Plasmodium spp*. coinfection on the outcome of EVD-related mortality. Random effects meta-analyses were conducted with the R package meta to produce overall proportion and effect estimates as well as measure between-study heterogeneity.

**Results:**

From 322 peer-reviewed papers, 17 were included in the qualitative review and nine were included in a meta-analysis. Prevalence of coinfection was between 19% and 72%. One study reported significantly lower coagulatory response biomarkers in coinfected cases but no difference in inflammatory markers. Case fatality rates were similar between EBOV(+)/Pl(+) and EBOV(+)/Pl(-) cases (62.8%, 95% CI 49.3–74.6 and 56.7%, 95% CI 53.2–60.1, respectively), and there was no significant difference in risk of mortality (RR 1.09, 95% CI 0.90–1.31) although heterogeneity between studies was high. One in vivo mouse model laboratory study found no difference in mortality by infection status, but another found prior acute *Plasmodium yoeli* infection was protective against morbidity and mortality via the IFN-γ signalling pathway.

**Conclusion:**

The literature was inconclusive; studies varied widely and there was little attempt to adjust for confounding variables. Laboratory studies may present the best option to answer how pathogens interact within the body but improvement in data collection and analysis and in diagnostic methods would aid patient studies in the future.

## Introduction

Viral epidemics have been recognised as an increasing global public health threat. As has been observed during the COVID-19, SARS and MERS outbreaks, our increasingly connected world allows rapid international spread of viruses like never before [1–5]. Spread of such pathogens to the African continent is of particular concern not only because of weak surveillance and health response systems, but also because of the high burden of other febrile infectious diseases, leading to problems with accurate diagnoses and patients afflicted with more than one infectious agent at a time [6]. Coinfections may alter immune responses and the expected course of infection, morbidity and mortality, perhaps requiring a different approach to case management among such individuals [7, 8].

Since malaria is highly prevalent across sub-Saharan Africa, it is reasonable to expect the prevalence of viral-P*lasmodium spp*. co-infection to be high in these settings. Understanding how *Plasmodium spp*. (herein *Plasmodium*) infection may alter responses to viral infection, and vice versa, is thus important for their treatment and control. Insights into this may be inferred from Plasmodium’s interaction with other established viral infections. Outbreaks of Ebola virus disease (EVD) occur sporadically in countries in Africa where there is already a high burden of malaria. Historically, the majority of EVD outbreaks have been relatively small with case numbers typically less than 100 people [6]. This, combined with the fact that the epidemics can progress very rapidly, occur in remote areas with dispersed populations, and that the affected countries already have weak disease surveillance systems and laboratory diagnostic facilities, means opportunities to investigate the epidemiology of EVD and other comorbidities were limited. The EVD outbreak of 2014 was massive in comparison, with 28,616 suspected, probable and confirmed cases and 11,310 deaths across the three majorly affected countries: Guinea, Liberia and Sierra Leone [6]. In 2013, the incidence of confirmed malaria cases in these three countries was 18, 280 and 279 per 1,000 population in Guinea, Liberia and Sierra Leone, respectively (though, this seems low for Guinea and was reported as 148/1,000 population in fiscal year 2014) [9, 10]. The huge local, national and international effort to control this EVD outbreak led to the collection of large amounts of data from health facilities and has allowed much greater investigation into the clinical and public health effects of Ebolavirus (EBOV) infection [11–13].

*Plasmodium* has previously been implicated in modulating the immune response to a secondary infection through activation of pro-inflammatory pathways [14–16]. In the case of respiratory viruses, this has a beneficial effect in preventing development of pneumonia in children, whereas it leads to uncontrolled bacterial growth and increased mortality with co-incident *Salmonella enterica* infection [17, 18]. *Plasmodium spp*. infection in HIV infected individuals leads to an increase in HIV viral load and could increase risk of HIV transmission and accelerate disease progression [19, 20].

EBOV and *Plasmodium* could interact in several different ways that would be of public health importance if EVD-related morbidity and/or mortality were reduced or increased. Despite the increase in data available since the West African EVD outbreak, to our knowledge, no one has attempted to synthesize available information on EBOV and *Plasmodium* coinfection and the overall effect on disease outcomes. There has been increasing threat of Ebola viral outbreaks in recent years yet still little is known about how co-morbidities such as malaria infection affect the Ebola immune response and disease outcomes. This, along with the advent of the Ebola vaccine, rVSV-ZEBOV-GP, means it is important to know how these infections may interact to optimise control and case management in the future [21]. With this in mind we set out to review the literature to answer: *How does Plasmodium co-infection affect the immune response*, *clinical profile and/or clinical outcome to Ebola virus infection*? We present a systematic review and meta-analysis of the literature to determine: 1) the prevalence of *Plasmodium* coinfection among cases of EVD in Africa, 2) the effect of EBOV/*Plasmodium* coinfection on EVD pathology and the immune response to EBOV infection, and 3) the impact of EBOV/*Plasmodium* coinfection on the outcome of EVD-related mortality. Finally, we discuss what gaps, if any, remain in the literature and what the implications are for case management and research in future EVD and other viral outbreaks.

## Methods

### Search strategy

The literature search was conducted with a final update on 15^th^ October 2020 by the lead study author in PubMed and Web of Science to look for published peer-reviewed papers with primary data on i) prevalence or seroprevalence of *Plasmodium* infection among individuals with current EBOV infection (cases of EVD) or with evidence of past EBOV exposure (indicated by seroprevalence), and/or ii) quantitative or qualitative data on the impact of EVD-related pathology and/or immune response, and/or mortality. To get a complete overview of the evidence we included for review epidemiological studies of infection in humans, clinical case reports and experimental laboratory studies using ex vivo and in vivo infection models. The search was conducted with the following search terms: (Ebola[Title/Abstract]) AND (malaria*[Title/Abstract] OR Plasmodium[Title/Abstract]). While this search strategy was very general, the number of returned articles was deemed feasible to screen without further narrowing of the search terms, thus ensuring all relevant papers could be identified. An initial screen of titles and abstracts excluded irrelevant papers based on being either: review papers, comments/editorials/correspondence, unrelated to EBOV or *Plasmodium* infection, research on vaccines or drug modes of action, not in the English language. Remaining papers were reviewed in their entirety and papers were included if they met the following criteria: 1) primary peer-reviewed research, 2) either i) epidemiological studies or genomic sequencing studies with explicit measurement of *Plasmodium* and EBOV co-infection prevalence and/or any measure of impact on EVD pathology/immune response/mortality, or ii) clinical case studies with disease pathology/immune response measures of co-infected patients, or iii) laboratory studies with ex vivo and/or in vivo models of EBOV and *Plasmodium* coinfection and a measure of effect on disease pathology and/or immune system modulation and/or mortality. Study of any *Plasmodium* or EBOV species and within any country and any date were included. In a subsequent meta-analysis, only epidemiological studies with prevalence of coinfection and CFR by malaria infection status and/or effect estimate data on the outcome of EVD-related mortality from countries in Africa were included. We did not include grey literature.

### Data extraction

All papers were included for qualitative review of methods and key findings. Observational cohort studies were also included for quantitative review in a meta-analysis. For the meta-analysis, data were extracted on the following variables: 1) study first author and publication details, 2) study design; 3) location and study period; 4) details of study population, including population size, mean age and sex breakdown; 5) source of data; 6) EVD-related data including method of diagnosis, EBOV species, number of EBOV positive (EBOV(+)) cases; 7) malaria-related data including method of diagnosis, Plasmodium species, number of sample that received a malaria diagnostic test, number of positive tests; 8) Mortality data including crude EVD case fatality rate (CFR), CFR of coinfected patients; 9) Effect estimates (e.g. risk ratios (RR), odds ratios (OR) and hazard ratios (HR)) for the effect of *Plasmodium* coinfection on EVD-related mortality; 10) other variables with a significant impact on mortality (potential confounders).

Extracted data were independently verified (i.e. double verification) by another member of the study team. Where necessary, study authors were contacted to verify study details. The authors conducted this meta-analysis in concordance with PRISMA standards of quality for reporting meta-analyses and the guidelines for Meta-Analyses and Systematic Reviews of Observational Studies [22, 23].

### Statistical analysis

We used a random-effects meta-analysis of proportions approach to quantify i) the prevalence of *Plasmodium* coinfection among EVD cases (EBOV(+)/Pl.(+)), ii) crude CFR of EVD cases, iii) CFR of EBOV(+)/Pl.(+) cases and iv) CFR of EBOV(+)/Pl.(-) cases. This was conducted using the metaprop function from the meta package in R [24, 25]. Metaprop allows specific analysis of binomial data and the computation of exact binomial and score test-based confidence intervals [26]. Metaprop was run with a random-effects model to account for heterogeneity between studies in terms of study population, differences in exposure and infection and diagnostic procedures. Random effects models assume the observed studies represent a distribution of possible effects and can incorporate both within-study variance and between-study heterogeneity. To conduct a meta-analysis with individual study weights, we used the inverse variance method of pooling with logit transformation and Clopper-Pearson 95% confidence intervals (CI). The prediction interval was calculated to give a range within which measurements of the same effect size would be expected to fall in future studies. Sub-group analyses were conducted where necessary and as described in the results (i.e. including Zaire EBOV (ZEBOV) species infections only).

For effect estimates related to the impact of *Plasmodium* infection on EVD mortality, where available, numbers of exposed (EBOV(+)/Pl.(+)) and unexposed (EBOV(+)/Pl.(-)) and with and without the outcome of death were used to calculate unadjusted RR and used in a random effects meta-analysis of RR using the metabin function within the meta package which allows meta-analysis of studies with comparison of two groups with binary outcomes [24]. Finally, any adjusted RRs reported in included studies were included in a meta-analysis using the metagen function which performs fixed and random effects meta-analysis based on estimates and their standard errors using inverse variance weighting for pooling [27].

Heterogeneity between studies was quantified for each proportion and effect estimate measure using the I^2,^ Chi^2^ and Tau-squared (*τ*^2^) statistics. I^2^ is a measure of the percentage of variability in the effect sizes that is not caused by sampling error, where 25% is considered low heterogeneity, 50% moderate heterogeneity and 75% substantial heterogeneity [28, 29]. Although I^2^ is not sensitive to the number of studies, it is sensitive to the precision of the included studies. Tau^2^ measures the between-study variance within a random effects model and is insensitive to the number and precision of included studies. The square-root of Tau^2^ is the estimated standard deviation of the underlying effects across all the studies [30]. In all models between-study variance was estimated using the Hartung-Knapp-Sidik-Jonkman (HKSJ) method, which is considered to be superior to the standard DerSimonion-Laird method in estimating variance of the pooled effect, particularly when the number of studies is small [31].

### Assessment of study quality and bias

Due to the small number of studies ultimately included in the effect size meta-analysis, publication bias via assessment of funnel plot asymmetry could not be assessed since the power to distinguish true asymmetry from chance is limited when the number of studies is <10 and is, therefore, not recommended [32]. However, studies included in the meta-analysis were assessed for quality using the Newcastle-Ottawa Scale, developed to assess the quality of nonrandomised studies incorporated into meta-analyses [33]. The scale uses a ‘star system’ to judge publications on three key areas: i) selection of study cohort(s), including representativeness of the exposed cohort and measure of, ii) comparability of cohorts based on whether important confounding factors were adjusted for in analyses, and iii) ascertainment of the outcome (for cohort studies) of interest including how it was assessed and whether rates of follow-up were sufficient to avoid potential study bias. In line with previous meta-analyses, included studies were assessed to be good, fair or poor based on the number of stars awarded within each category: “good” meant 3 or 4 stars in selection, 1 or 2 stars in comparability, and 2 or 3 stars in outcomes; fair meant 2 stars in selection, 1 or 2 stars in comparability, and 2 or 3 stars in outcomes; and “poor” meant 0 or 1 star(s) in selection, or 0 stars in comparability, or 0 or 1 star(s) in outcomes. Thus, studies had to achieve at least one star in each section to be deemed good or fair.

## Results

### Search results

**Fig 1:**
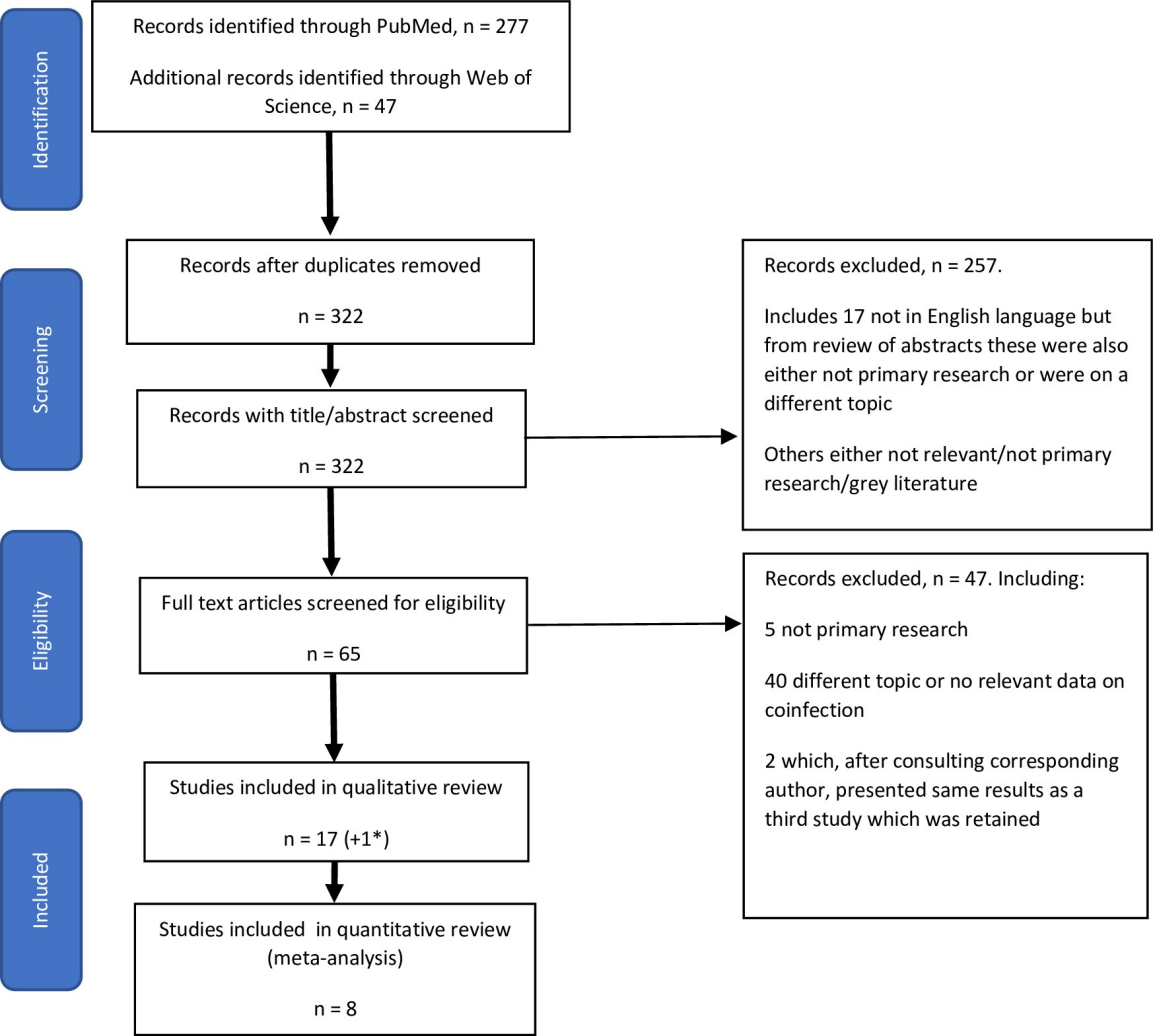
PRISMA flow diagram for study selection.

Following removal of duplicates, the initial search returned 322 results which had titles and abstracts screened for eligibility (Fig 1). Following the initial screen, 65 results were reviewed in their entirety and a final 17 papers were included for qualitative review as they had a measure of EBOV and *Plasmodium* coinfection prevalence and/or impact on disease outcome according to our inclusion criteria (Table 1). An additional paper was retained since it presented additional demographic information on the same cohort of patients as another which presented coinfection outcome data [34, 35]. We noted potential overlap of the study population between some studies which were confirmed with study authors. Since the studies had different sample sizes, methods and results, all were retained for review and the overlap of populations clearly marked in Table 1. Overlapping studies were excluded in the quantitative meta-analysis so that the study with the largest study population was included in each instance.

**Table 1:** Details of studies included in the qualitative and quantitative reviews.

Observational studies											
Population Id	Study #	Publication	Study design	Study location and study period	Study Population	Source(s) of exposure and outcome data	EVD data	Malaria data	Outcome data	Effect estimate (unadj. and adj.)	Included in meta-analysis?
1	1	Barry et al. Med Mal Infect. 2014; 44(11–12):491–4	Prospective health facility-based cohort	Conakry, Guinea Mar-Aug 2014	Patients with confirmed EVD (by RT-PCR) admitted to a single ETU (N = 90) Demographics • Male: 63% (n = 57) • Mean age: 34 ± 14 yrs (range of age groups 1–20 years to 61+ years) • Days from symptom onset to hospitalisation: 6 ± 3 days	Data collected prospectively by trained medical staff	EVD(+) cases: 90 Diagnostic: RT-PCR EBOV species: Zaire Viral load: N/A	*Plasmodium* infection among EVD(+) cases: Tested = 90 Positive = 65/90 (72%) Diagnostic: not reported Species: unspecified Parasite load: N/A	Crude CFR: 39/90, 44% (95% CI 33–54) CFR of coinfected cases: N/A	N/A	No—CFR by malaria status not reported, just prevalence of malaria co-infection
2	2	Kratz et al. PLoS One. 2015; 10(6):e0129333	Prospective health facility-based cohort	Isiro, DRC Aug-Nov 2012	Probable & confirmed EVD cases (N = 52) either: 1. admitted to a single ETU in Isiro (N = 18) 2. identified in the community (N = 34) Demographics • Male: 23.1% (n = 12), incl. 38.9% ETU cases (n = 7),14.7% community cases (n—5) • Age: 15-54yrs overrepresented in community cases; ≤14yrs underrepresented in community and ETU cases • 61% ETU cases admitted within 3 days of symptom onset	Demographic and clinical data obtained from routine data collected by ETU staff	EVD(+) cases: 52 • 18 ETU patients (all confirmed EVD(+)) • 34 community cases (16 probable, 18 confirmed EVD+)) Diagnostic: PCR or IgM-ELISAEBOV species: BDBVViral load: N/A	*Plasmodium* infection among EVD(+) cases: Tested = 9 Positive = 4/9 (44.4%, three positive RDTs and one treated as presumptive malaria) Diagnostic: Bioline RDT (unspecified if Pf only or Pf/pan) Species: unspecified Parasite load: N/A	Crude CFR: 28/52, 53.8% Including: CFR of community cases: 55.9% (93.8% probable cases, 22.2% confirmed cases) & CFR of ETU admissions: 50.0% (all confirmed cases) Concomitant malaria did not influence CFR (CFR 50% with and without malaria, raw numbers not provided)	N/A	Yes—prevalence and CFR by malaria status reported
3	3	Gignoux et al. New Engl J Med. 2016. 7;374(1):23–32	Retrospective health facility-based cohort	Foya, Liberia Jun-Oct 2014	Confirmed EVD patients admitted to single ETU (N = 381) Demographics • Male: 48.3% (n = 184) • Age (years): 0–4 = 26 5–29 = 186 30–59 = 144 ≥60 = 22 • Days from symptom onset to admission = 3.5 (IQR 2–6)	Data compiled by staff epidemiologist from case-investigation forms, clinical files and lab results	EVD(+) cases: 381 Diagnostic: qRT-PCR EBOV species: Zaire Viral load, median PCR Ct at admission: 19.4 (IQR 17.1–22.8)	*Plasmodium* infection among EVD(+) cases: Tested = 337 Positive = 65/337, 19.3% Diagnostic: BinaxNOW RDT Species: unspecified although RDT detects all four primary species Parasite load: N/A	Crude CFR: 202/328, 61.6%, (53 cases with missing outcome data) CFR of coinfected patients: 34/56, 60.7%, CFR of EVD(+)/*Plasmodium*(-): 144/239, 60.3%	Increased risk of death with *Plasmodium* coinfection:Unadj.RR = not reportedaRR = 1.23, 95% CI 0.53–2.86, p = 0.63(adjusted for antimalarial prescription, age group, sex, Ct value at admission, days from symptom onset to admission, receipt of IV fluids, # inpatients at Ebola treatment centre on day of admission)Risk of death also assoc. with: • Antimalarial treatment: ASAQ treatment associated with lower risk of death than A-L, aRR 0.69, 95% CI 0.54–0.89 • age, days from symptom onset to admission, IV fluids and # inpatient Stratified analysis showed malaria infection may inhibit the response to drug treatment—ASAQ was protective in malaria(-) but not in malaria(+) patients:aRR ASAQ treatment group compared to A-L reference group:Malaria (+) = aRR 1.00 (0.54–1.85), p = 0.98Malaria(-) = aRR 0.64 (0.49–0.85), p = 0.002	Yes—prevalence and CFR by malaria status reported and effect estimate
4	4	De Wit et al. Emerg Infect Dis. 2016. 22(2):323–6.	Retrospective cohort/Diagnostic feasibility	Monrovia, Liberia Oct 2014-Mar 2015	Samples from suspect EVD patients submitted to single diagnostic laboratory (N = 1,058) Demographics not reported	Data from CDC-NIH ELWA diagnostic laboratory	EVD(+): 306/1058 Diagnostic: RT-PCR Species: Zaire Viral load: N/A	*Plasmodium* infection overall sample: Tested = 1058 Positive = 290 EVD(+) cases with *Plasmodium* Coinfection: 47/306 (15.4%) Diagnostic: qRT-PCR (Ct ≤ 30 considered positive; used to make comparable to sensitivity of microscopy) Species: 95% Pf, all-species *Plasmodium* included in analysis Parasite load, mean Ct by EBOV infection status: EBOV(+) 24.7 EBOV(-) 20.37 (p<0.01)	N/A	N/A	No—overlap of study population with Rosenke et al. and no CFR reported
4	5	Rosenke et al. Clin Infect Dis. 2016. 63(8):1026–33	Retrospective health facility-based cohort	Monrovia, Liberia Aug 2014-Feb 2015	EVD(+) confirmed cases admitted to single ETU (N = 1182) Demographics • Male: 48.3% • Mean age: 29.8 ± 15.6 (range 0.01–83) • <5 years not included in effect size estimates due to low numbers	Demographic and clinical data sourced from patient data forms submitted to diagnostic lab and updated with lab test results Outcome determined by cross-referencing names on patient data forms with a list of deceased patients at ETU	EVD(+) cases: 1182 Diagnostic: qRT-PCR Species: Zaire Viral load, mean Ct ± SD: 27.0 ± 4.7	*Plasmodium* infection among EVD(+) cases: Tested = 956 Positive = 185/956, 19% positive Dx: qRT-PCR, Ct ≤ 30 considered positive Parasite load, mean Ct ± SD: 24.8 ± 4.0	Crude CFR: 612/1182, 51.8%Coinfected CFR:78/185, 42.2%CFR EBOV(+)/Pl(-): 54%(P = 0.007)CFR by *Plasmodium* Ct level: • Ct < = 20: 5/29, 17.2% • Ct>20: 73/156, 46.7%P = 0.007Other significant predictors: Age, sex, EBOV viral loadViral load changed effect of *Plasmodium*: • High viral load = no effect of *Plasmodium* infection • Moderate-to-low viral load (Ct≥25) = *Plasmodium*(+) versus *Plasmodium*(-) survival rate 94% vs 54% ages 5–39 years, and 100% vs 41% for ages >40yrs, p<0.0001	RRs reported as effect on survival. *Plasmodium* coinfection associated with increased survival: Unadj. RR = not reported aRR 1.2 (1.1–1.4, p = 0.004) aRR for survival from EVD stratified by *Plasmodium* Ct level: EVD(+)/Pl.(-): comparison group High *Plasmodium* burden (Ct < = 20) = RR 1.4, 1.2–1.7, p<0.0001 Low *Plasmodium* burden (Ct>20) = RR 1.2, 0.98–1.4, p = 0.08 aRR adjusted for: age, sex and EBOV Ct level	Yes—prevalence and CFR by malaria status reported and effect estimate
5	6	Kerber et al. J Infect Dis. 2016 Oct 15; 214(Suppl 3): S250-S257	Prospective health facility-based cohort	Guinea (cases mostly from Macenta and Guéckédou)	Suspect EVD cases attending hospitals/ETUs in Guinea (N = 2178) and community deaths (N = 563) with samples submitted to EMLab unit in Guéckédou, Guinea Demographics • Male: 47% suspect hospital cases, 52% community deaths • Median age (IQR): 30 (18–44) suspect hospital cases, 37 (25–55) community deaths	Laboratory request forms uploaded to EMLab database—cross-referenced with Guinean EVD patient database	EVD(+) confirmed: 1231/2178 suspect cases (57%), 281/563 community deaths (50%)Diagnostic: qRT-PCRSpecies: ZaireViral load (median, IQR): • Hospitalised and survived 23.2, 20.4–26.5 • Hospitalised and died 18.1, 16.2–20.8 • Community deaths 21.5, 19.4–24	*Plasmodium* prevalence overall: 1937/2178 (89%) hospital attendees Coinfection prevalence among hospital EVD cases: 261/1231 (24%) *Plasmodium* coinfection highest children aged <15years Diagnostic: BinaxNOW RDT (detects Pf/pan) Species: unspecified although RDT can detect all four major species	Crude CFR: 719/1205, 59.7% among hospitalised EVD patients Mortality rate highest among patients aged < 5 years and >74 years (80 and 90% respectively). CFR by infection status: EBOV(+)/Pl.(+): 150/249, 60.2% EBOV(+)/(Pl.(-): 452/798, 56.6% Malaria co-infection increased CFR in 5-14yr age group by >20%: EBOV(+)/Pl.(+): 43/69, 62.3% EBOV(+)/Pl.(-): 22/54, 40.7%	Odds of fatality assessed among 1047 EVD(+) patients with complete datasets Crude OR for fatal outcome: EBOV(+)/Pl.(-): reference EBOV(+)/Pl.(+): 1.2 95% CI 0.9–1.5, p = 0.32 OR for fatality with interaction between *Plasmodium* infection and age, among 5-14yrs age group: EBOV(+)/Pl.(-): reference group EBOV(+)/Pl.(+): 4.2 95% CI 1.7–10.1, p = 0.002 No significant effect observed at any other age group, adjusted for EBOV Ct value. Other sig variables: EBOV Ct value	Yes—prevalence and CFR by malaria status reported and effect estimate
5	7	Carroll et al. mSphere. 2017 2(4):e00325-17	Metagenomic RNA Deep-sequencing study, retrospectively sampled patient cohorts	Guéckédou, Guinea	EVD cases diagnosed by European Mobile Laboratory in Guéckédou, Guinea including hospitalised patients and deaths in the community N = 202, incl: • 44 Hospitalised survivors (29.5% male, mean age 33.1yrs, Mean EBOV Ct 21.7 (range 15.8–31.6)) • 118 Hospitalised fatalities (40.1% male, mean age 28.4yrs, mean EBOV Ct 17.2 (range 12.1–26.6)) • 24 Community deaths with swabs for microbiome analysis (29.1% male, mean age 33.4yrs, Mean EBOV Ct 18.8 (range 12.4–31.9)) • 16 Patients convalescent for EVD and RT-PCR negative (93.75% male, mean age 30yrs, mean EBOV Ct N/A) Immune system marker analysis conducted on: EBV+/Pf+ (n = 10), and EBV+/Pf- (n = 13)	Clinical patient records Blood and plasma samples sequenced using HiSeq 2500 system	EBOV infection: Reads mapped to Zaire ebolavirus in all samples	*Plasmodium* infection: RDT results (from patient records) = 40/121, 33% Sequenced reads = 156/186, 84% blood and plasma samples contained reads mapping to Pf Read depth varied widely (FPKM value as a measure of read depth varied from 59 to 358820)	Using RDT data alone—*Plasmodium* increased patient mortality (p = 0.053, ANOVA) Using sequencing data—Patients experiencing the highest burden of *Plasmodium* infection had a mortality rate of 87% (n = 6), higher than patients with low parasite burden Mortality increased with *Plasmodium* sequence read depth, but no absolute correlation observed Immune system markers associated with severe malaria in absence of EBOV were compared between EBOV(+)/Pf(+) (n = 10) and EBOV(+)/Pf(-) (n = 13) patients: *Plasmodium* infection had no additional effect on the innate immune response to EBOV, but markers associated with the coagulation pathway were increased in patients that were negative for Pf infection.	N/A	No—overlap of study population with Kerber et al.
6	8	Hartley et al. PLoS Negl Trop Dis. 2017. 11(2): e0005265/e0005356*	Retrospective health facility-based cohort	Port Lko, Sierra Leone Dec 2014-Nov 2015	Suspect EVD patients admitted to a single ETU (GOAL-Mathaska ETU) (N = 566) Demographics: • Mean age 32.4yrs, incl. 30.6 EVD(+), 33.1 EVD(-) • 52% Male overall sample, 48% Male EVD(+) • Referral time among EVD(+), mean: 4.2 days	Routine clinical files	EBV diagnosed in 27.9% patients (n = 158/566) Dx: semi-quantitative RT-PCR Species: Zaire Viral load, mean Ct (range): 22.0 (13.5–37.9); and 39% EVD(+) cases with Ct<20	*Plasmodium* infection in overall sample: Tested = 543/566 Positive = 188, 34.6% *Plasmodium* coinfection among EVD(+) cases: Prevalence = 35/145, 24.1% 2x greater odds of malaria infection among EVD(-) than EVD(+): 38% v 24%, OR = 2, p = 0.005 Prevalence of *Plasmodium* infection highest in <5 yrs Diagnostic: HRP-II RDT (detects Pf only) Species: unspecified but only Pf diagnosed Parasite load: N/A	CFR: Crude = 96/158, 60.8%, EBOV(+)/Pf(+) = 26/35, 74.3% EBOV(+)/Pf(-) = 53.6%	OR for fatality: EBOV(+)/Pf(-) = reference EBOV(+)/Pf(+) = 3.9, p = 0.03 (adjusted for age and sex) However, co-infected patients had significantly higher viral loads than those with EVD alone (mean Ct 20.8 vs. 22.3, p<0.01) and after controlling for viral load, the increased mortality among co-infected patients disappeared (p = 0.1). CFR sig. associated with age, certain symptoms, referral time and viral load (Ct ≤ 20 assoc. with 12.6x higher odds of fatality compared to Ct > 20 (p<0.0001))	Yes—prevalence and CFR by malaria status reported and effect estimate
7	9	Vernet et al. JCI Insight. 2017. 2(6):e88864	Prospective health facility-based cohort	Macenta and Nzerekore, Guinea Nov 2014-jan 2015	Suspect EVD patients admitted to two ETUs (N = 168) Clinical follow-up on 77 EVD(+) patients Biochemical analysis on 67 EVD(+) patients and EVD(-) febrile controls Demographics of EVD(+) cases (N = 97): • Male 36% (n = 35) • Age groups, n: 0–9 = 11 10–19 = 19 20–44 = 45 ≥45 = 22 • Time between symptom onset to admission: ≤3 days 31%, >3 days 69%; mean 4.6 days (range 1–14)	Clinical data from ETU patient records Biological data from Pasteur laboratory in Macenta	EVD(+) patients: 97/168 (58%)Diagnostic: RT-PCR, Ct ≤ 34 considered positiveViral load, baseline viraemia arbitrary units (AU, defined as AU = 2^(34—Ct)^): • <25,000 AU (equivalent to Ct 19.5) = 55% (n = 41) • ≥25,000 = 45% (n = 33)	*Plasmodium* infection among EVD(+) cases: Tested = 74 Positive = 14/74, 18.9% Diagnostic: ONSITE Pf/pan RDT. Species: *P*. *falciparum*	Crude CFR: 57/97, 58.7% CFR EVD(+)/Pf(+): 12/14, 85.7% CFR EVD(+)/Pf(-): 36/60, 60.0% (p = 0.12)	CFR significantly assoc. with baseline viremia	Yes—prevalence and CFR by malaria status reported
8	10	Smit et al. J Infect Dis 2017. 64(3):243–249	Retrospective health facility-based cohort	Liberia and Sierra Leone 5 ETUs run by IMC Sep 2014-Sep 2015	Children <18 years with confirmed EVD (by qRT-PCR) and outcome data at 5 ETUs (N = 122) Demographics: • Median age 7yrs • Male 44% (n = 54)	Clinical data forms digitized into unified database and linked with laboratory data and malaria testing	EVD(+) cases: 122 Diagnostic: qRT-PCR, Ct ≤ 40 considered positive Species: Zaire Viral load, median Ct (IQR): survivors 25 (21.3–27.5); died 19.2 (16.9–23.7), p < .001	Malaria testing available from Sierra Leone only. *Plasmodium* infection among EVD(+) cases: Tested = 68/84 Positive: 27/68, 40% Diagnostic: BinaxNOW Species: unspecified although diagnostic distinguishes 4 major species Parasite load: N/A	Crude CFR 69/122, 56.6%	CFR significantly associated with age, bleeding, median initial EBOV Ct, length of hospital stay *Plasmodium* parasitaemia had no impact on EVD outcomes in aggregate (p = 0.76) or age-stratified analyses (data not shown)	No—overlap of study population with Waxman et al., children aged <18 years only
8	11	Waxman et al. 2017. 17(6):654–660	Retrospective health facility-based cohort	3 ETUs in Lunsar, Makeni and Kambia (run by IMC), Sierra Leone Dec 2014-Oct 2015	Suspect EVD patients admitted to three ETUs with test results for both EBOV and *Plasmodium* infection (N = 1368) Demographics: • Age, median (IQR): overall = 29yrs (20–44) EBOV(+)/Pl.(-) = 30yrs (20–45) EBOV(+)/Pl.(+) = 13yrs (4–27) • 53% (n = 715) Male in overall sample; 36.1% (n = 35) Male in EVD(+) cases alone	Clinical paper forms filled in by medical care staff and uploaded to single database	EVD diagnosed in 254/1368 patients (19%) Dx method: RT-PCR (Ct<40) Species: Zaire Viral load: N/A	*Plasmodium* infection: Overall sample tested for malaria = 1368 (100%) Prevalence of malaria: EBOV(+) = 53/254, 21% EBOV(-) = 365/1114, 33% Diagnostic: BinaxNOW RDT Species: unspecified although RDT distinguished all four major species Parasite load: N/A	CFR: Crude = 140/254, 55.1%, 95% CI 48.8–61.3% EBOV(+)/Pl.(+) = 66%, 35/53 EBOV(+)/Pl.(-) = 52%, 105/201 28-day mortality highest in co-infected patients (8.2 per 100 person-days, 95% CI 5.9–11.4)	Cox proportional hazards model for effect on mortality: • EBOV(-)/Pl.(-) = reference group • EBOV(+)/Pl.(+) aHR = 9.4, 6.2–14.2, p<0.0001 • EBOV(+)/Pl.(-) aHR = 6.0, 4.4–8.0, p<0.0001 • EBOV(-)/Pl(+) aHR = 0.4, 0.2–0.7, p = 0.001 Subgroup analysis of EVD(+) cases only by *Plasmodium* infection:Pl.(-) aHR = reference groupPl.(+) aHR = 1.69, 1.14–2.52, p = 0.00917/10 symptoms differed by infection status—fever, headache, abdominal pain, emesis, diarrhoea, arthralgia or myalgia, dyspnoea	Yes—prevalence and CFR by malaria status reported and effect estimate
8	12	Garbern et al. Open Forum Infect Dis. 2019. 6(7):ofz250	Retrospective health facility-based cohort	5 ETUs run by IMC, Liberia & Sierra Leone Sep 2014-Sep 2015	EVD(+) patients admitted to 5 ETUs (N = 424) Demographics • Median age 30yrs (16–44) • 40.6% Male	Data obtained from standardised clinical records forms	EVD cases: 424 Diagnostic = qRT-PCR, Ct < 40 considered positive Viral load: Ct ≤ 22 = 159, 37.5% Ct > 22 = 122, 28.8% missing = 143, 33.7% Among subset with data available: Ct ≤ 22 = 56.6%	*Plasmodium* infection data from whole sample: Tested = 243/424, 57% Positive = 48 (11.3%) Negative = 195 (46.0%) Missing = 181 (42.7%) From tested subset: Positive = 48/243, 19.8% Diagnostic: BinaxNOW RDT Species: unspecified Parasite load: N/A	Crude CFR: 244/424, 57.5 EVD(+)/Pl.(+) CFR: 31/48, 64.6% EVD(+)/Pl.(-) CFR: 101/195, 51.8% EVD(+)/Pl.(missing) CFR: 112/181, 61.9% Malaria RDT result in survivors vs. died: - 9.4% v 12.7% positive - 52.2% v 41.4% negative - 38.3% v 45.9% missing	Covariates significantly associated with mortality = time to ETU opening, Ct value, abnormal bleeding, diarrhoea, dysphagia and dyspnoea Insignificant difference in mortality by *Plasmodium* infection status (p = 0.08) Some patients assumed exposed to ASAQ MDA showed non-significant decrease in risk of death—RR 0.63, 0.37–1.07, p = 0.086	No—overlap with Waxman et al.
9	13	Li et al. J Clin Microbiol. 2019. 57(9):e00827-19.	Metagenomic RNA NGS study, retrospectively sampled cohort	DRC	Suspect EVD patients from Boende, DRC Aug 2014-Sep 2014 N = 70 Including 37 confirmed/probable EVD cases with demographics: 5/37 (13.5%) Male Mean age 35.4yrs (±16.1)	Samples tested by RT-PCR in DRC, then shipped to US for repeat RT-PCR and mNGS followed by EBOV-specific capture probe	EVD(+) cases: • 22 confirmed (at least 2/3 positive tests) • 15 probable (1/3 positive tests) Breakdown of EBOV prevalence by test:RT-PCR in DRC = 31/70 (64.5%)RT-PCR in US = 21/70 (30%)mNGS reads = 22/70 (31.4%)EBOV reads—mean 8,099, SD ± 41,246 (range 1–286,723)	Pf data: RNA reads in 21/70 (30.0%) patients, number of reads varied between 1 and 248,696 per sample, mean 4548 ± SD 29,980 Coinfected samples: 9/37 confirmed/probable EVD(+) cases coinfected with Pf (24.3%)	CFR overall: 38/70 patients CFR EVD(+) cases: 23/35, 65.7% (2 missing outcome data) CFR by *Plasmodium* infection status for 25 patients with clinical data available: EBOV(+)/Pf(-) = 10/18, 55.6% EBOV(+)/Pf(+) = 5/7, 71.4% P = 0.28 No significant differences in clinical characteristics (e.g. fever, headache, vomiting ETU.) were found when comparing EBOV(+)/Pf(+) (n = 7) to EBOV(+)/Pf(-) (n = 18) Thus, no significant effect of coinfection on disease severity or mortality but only small sample size	N/A	Yes—prevalence and CFR by malaria status reported and effect estimate
10	14	Abbate et al. Emerg Infect Dis. 2020. 26(2):229–237.	Cross-sectional survey	Gabon Survey conducted between July 2005—May 2008	Permanent residence aged >15years from 210 rural villages (population < 300) across 9 administrative provinces N = 4272 enrolled, malaria and EBOV status obtained from 4170 incl.: 47.3% male median age 49	Random sampling of villages, stratified by province—each province surveyed once during field missions from July 2005-Mat 2008, generally during dry season Other population-level indicators taken from 2003 national census data, and Demographic and Health Survey 2012	ZEBOV-specific IgG antibody seroprevalence 638/4170, 15.3% Diagnostic: ZEBOV-specific IgG ELISA for seroprevalence Species: Zaire	*Plasmodium* infection in whole sample: 52.5% infected with 1 or more *Plasmodium* species *Plasmodium* coinfection in seropositive EBOV = 425, 10.2% Significant overabundance of coinfection—χ^2^ = 59.4, df = 1, p<0.0001 Diagnostic: in-field blood smear & high-throughput targeted sequencing of *Plasmodium*-specific cytochrome b mitochondrial DNA (able to distinguish species, single and mixed infections) Species: sequencing identified Pf, Pm and Po infections; all-species infection used for main analysis	CFR: N/A	Due to missing data, analysis of individual risk factors conducted on 3912 persons: Positive correlation between geographic distributions (prevalence across administrative departments) of EBOV exposure and *Plasmodium* positivity (Spearman rank correlation coefficient ρ = 0.43, df = 42, p<0.01) Individual level = prior exposure to Ebola strongly associated with increased probability of current *Plasmodium* spp. infection even after accounting for geographic locations and all other individual and population level risk factors (aOR = 1.741 (1.40–2.14) p<0.0001) EBV antibodies was stronger risk factor for *Plasmodium* infection than any other individual trait and second only to living in a Lakeland habitat. Associations for all-species *Plasmodium* were qualitatively identical to Pf and Pm considered separately (Po infection too rare)	No—no measure of current EBOV infection
**Experimental laboratory studies**											
	**Study #**	**Study**	**Experimental design**		**Methods**		**Key results**				
	15	Rosenke et al. 2018. J Infect Dis. 218(suppl 5):S434-S437.	Murine model of coinfection, time-course studyModel details: • CD1 mice • Model of human *Plasmodium* infection = Py (strain 17XNL), nonlethal in mice • Model of human EBOV infection = mouse-attenuated EBOV (MA-EBOV)		Eight groups of 10 mice with intraperitoneal inoculation of Py (10^4^ parasitized erythrocytes), followed by intraperitoneal inoculation of MA-EBOV on different days post Py inoculation (dpi): One group on dpi-0 and then one group on each third dpi (i.e. dpi-3, dpi-6…to dpi-21) Single high dose of MA-EBV administered: MA-EBV 100 median lethal dose 6 mice per group used to observe survival, 4 scheduled for necropsy on day 4 post MA-EBOV inoculation Outcome: survival over time No immune system markers measured		One mouse infected on dpi-0 survived but all other mice died. No statistically significant differences were observed between groups in terms of RBC number, MA-EBOV RNA levels. Repetition of dpi-0 time point with 40 coinfected mice observed no beneficial effect				
	16	Rogers et al. Cell Rep 2020. 30(12):4041-4051.e4.	Ex vivo model of coinfection: • Naïve human and mouse macrophages • *Plasmodium* infection: Challenge with sera from acutely parasitaemic rodents • EBOV infection: non-human primate sera from rhesus macaques infected with *P*. *cynomolgi* In vivo murine model of coinfection: • Mice: BALB/cJ, C57BL/6 IFN-αβ receptor-null mice (Ifnar^-I-^), wild-type C57BL/6, and C57BL/6 IFN-γ-receptor-null (IfngrI^-I-^) • *Plasmodium* infection: Py (& Pcc) • EBOV infection: MA-EBOV, rVSV/EBOV GP, WT-EBOV (all Mayinga, a variant of Zaire EBOV)		In vivo experiments: 6 groups 10 mice • Day -6 relative to MA-EBOV exposure, 3 groups inoculated with 10^6^ Py-infected RBCs • Day 0 challenged with 1, 10, or 100 plaque-forming units of MA-EBOV intraperitoneally • Day 3—three mice each group euthanised to determine viremia and viral load, others observed up to 18 days • Lower EBOV doses repeated with 4 additional groups of 10 mice • KO mice followed protocol as above but challenged with rVSV/EBOV GP on Day 0 instead of MA-EBOV • both Py and Pcc infection tested but Pcc discontinued Ex vivo experiments: Macrophages challenged with serum from P. cynomolgi-infected rhesus macaques followed by rVSV/EBOV GP exposure Outcomes: mortality, viremia and viral load (spleen, liver, kidneys)		• Py-infected mice challenged with a low, but lethal, 1-iu MA-EBOV dose showed reduced morbidity and mortality (P = 0.0076). These mice had 3-log reduction in viral titres on day 3, and a 1- to 2-log reduction in viral load in liver and spleen. These mice also had 15- to 260-fold lower viremia over first 60hours post viral infection and lower viral load in spleens, livers and kidneys • Challenge with MA-EBOV at doses of 10-iu and 100-iu produced a reduced protective effect • Py and Pcc-infected Ifnar^-l-^ mice were protected from lethal dose of rVSV/EBOV GP, showing no role of type 1 IFN signalling in conferring protection • IFN-γ was elevated in Ifnar^-l-^ and WT mice after Py infection • Similarly, human macrophages exposed to P. cynomogli-inoculated serum had 20- to 30-fold lower viral replication & elevated M1 markers assoc. with IFN- γ signalling • Ifnar^-l-^IfngrI^-I-^ mice infected with Py had no protection from rVSV/EBOV GP over Py-uninfected mice, although, these KO mice did produce elevated IFN- γ when exposed to rVSV/EBOV • T cells but not NK cells were required for Py-mediated protection • Administration of single high-dose recombinant IFN- γ only protected mice within 24hours of rVSV/EBOV challenge, suggesting more persistent, sub-patent *Plasmodium* infection and/or sustained, low-grade IFN- γ production offers protection more strongly than single bolus of cytokine • Protective effect waned over time with Py-infected mice protected from EBV challenge three weeks after Py infection, but not by 5 weeks.				
**Clinical case reports**											
	**Study #**	**Publication**	**Locations**		**Patient details & methods**		**Relevant outcomes/findings**				
	17	Muehlenbachs et al. 2017. 215(1):64–69.	Patient 1 from Gulu, Uganda Patient 2 from Isiro, DRC		Two pregnant EVD(+) patients: • Gulu, Uganda in 2000 (Sudan virus) • Isiro, DRC in 2012 (BDBV virus) EBOV diagnostic: • SUDV RT-PCR & ELISA in Gulu • BDBV RT-PCR in Isiro Placenta, foetal tissue and post-mortem skin biopsies performed and processed using standard histological methods		Patient 1: Infected with Sudan virus Outcome: spontaneous still birth, mother recovered Analysis of placenta: • moderate malarial parasite pigment in fibrin and within macrophages embedded in fibrin • immunohistochemical analysis showed EBOV antigen in placenta, primarily within areas of fibrin deposition, localised to maternal mononuclear cells including malarial parasite pigment-laden macrophages Patient 2: Infected with BDBV Outcome: spontaneous live birth followed by infant and maternal death Analysis of placenta: • evidence of viral inclusions but no malarial parasite pigment				

*Two publications from Hartley et al. in relation to same cohort—PLoS Negl Trop Dis 11(2)e0005355 is source of prevalence and CFR data, PLoS Negl Trop Dis 11(2):e0005356 provides extra demographic info.

The final set of papers qualitatively reviewed thus included 14 observational studies (13 cohort studies and one cross-sectional study), two laboratory studies and one clinical case report. A final set of nine cohort studies were included in the quantitative meta-analysis based on having a unique cohort of patients with similar patient characteristics. Studies were excluded if drawn from the same sampled cohort as another more up-to-date or larger study (n = 3), or if they sampled only children (n = 1).

#### Cohort studies

Nine retrospective cohort studies [34, 36–43] and four prospective cohort studies [44–47] were identified, all with health-facility based cohorts of suspect and/or confirmed EVD cases from Ebola treatment units (ETUs) in Liberia (n = 3), Sierra Leone (n = 2), DRC (n = 2), Guinea (n = 4), and with sites in both Liberia and Sierra Leone (n = 2) although these only included malaria testing in Sierra Leone. Due to overlap of study populations, however, these 13 studies drew from nine unique populations (as indicated in Table 1).

Sample size of the study cohorts varied from 52 to 2741 and EVD cases from 52 to 1512. Twelve of the studies included all age groups in their cohort, while one included only children aged <18 years. Percentage of males among EVD(+) cohorts was mostly between 44 and 52% (n = 5), although ranged from 13.5% to 63%.

EVD was diagnosed by real-time PCR (RT-PCR) in eleven cohort studies including seven studies [34, 36, 38, 40, 42, 46, 47] which used quantitative RT-PCR (qRT-PCR) through which cycle threshold (C_t_) was analysed as a proxy for viral load. Cycle threshold is defined as the cycle number of the assay at which the fluorescence exceeds a certain threshold, with lower Ct values indicative of higher viral load and vice versa [48]. The remaining two studies utilised a metagenomics (mNGS) approach to identify EBOV RNA in blood and plasma samples from EVD patients. One of these used a combination of RT-PCR test data from both DRC and US-based laboratories and mNGS results to determine whether patients were confirmed (at least two positive tests) or probable (one positive test) EVD cases. Although rarely explicitly stated, from the outbreak date and location, most of the observational studies were inferred to be investigating *Zaire ebolavirus* (ZEBOV, n = 12); only one investigated *Bundibugyo ebolavirus* (BDBV).

Malaria diagnosis was conducted by rapid diagnostic test (RDT) in most of these studies except two in which malaria diagnosis was made by qRT-PCR, with Ct ≤ 30 indicative of a positive *Plasmodium* infection considered to provide equivalent sensitivity to microscopy [37, 38]. These two studies did, however, have overlap of the study population and only one explored the effect of parasite load on mortality-related outcomes using Ct level as a proxy [38]. Another two studies identified RNA reads mapping to *Plasmodium* to determine malaria infection prevalence.

*Plasmodium falciparum* (Pf) was specified as the causal agent of malaria in one cohort study [47] and one other estimated 95% of malaria infections in their sample were *Pf* although subsequent analyses included all species [37]. The mNGS studies also identified *Pf* as the causal agent in their samples. The rest did not specify Plasmodium species in their analyses but all used RDTs that detect all four major Plasmodium species (Pf, *Plasmodium vivax* (Pv), *Plasmodium malariae* (Pm) and *Plasmodium ovale* (Po)), except one which used a HRP2 RDT detecting Pf only [34].

Only one study [39] attempted to look at signalling/response pathways between EBOV(+)/Pf(+) and EBOV(+)/Pf(-) patients in an attempt to discern any immune response modulation, and one other compared clinical symptoms between the two infection groups [43].

#### Cross-sectional studies

One cross-sectional national survey was identified in Gabon which covered a general rural population of 4,272 and measured past exposure to ZEBOV infection using seroprevalence of ZEBOV-specific IgG antibodies, and current Plasmodium spp. infection using in-field blood smear and Plasmodium mitochondrial DNA sequencing. DNA sequencing identified Pf, Pm and Po infections within the sample but main analyses included all-species Plasmodium but was also reported for Pf and Pm infections only.

#### Clinical case reports

One clinical case report was identified describing the pathology of EVD and malaria coinfection during pregnancy. The clinical case report included one pregnant woman from Gulu, Uganda admitted with Sudan virus in 2000, and a second from Isiro, DRC, infected with BDBV in 2012. EVD was diagnosed using species-specific RT-PCR in each location as well as ELISA in Gulu. Histological methods were employed to analyse EVD and malaria pathology in placental and foetal tissues following delivery.

#### Laboratory studies

Both laboratory studies used an in vivo murine model of coinfection to analyse the impact of prior *Plasmodium* infection on outcomes related to EVD. The model in one study was of CD1 mice infected via intraperitoneal inoculation with *Plasmodium yoeli* (Py) at a dose of 10^4^ parasitized erythrocytes, followed by challenge with mouse-attenuated EBOV (MA-EBOV) at a 100 median lethal dose at different time points post Py infection. In each time-group, 6 mice were followed for survival to 28 days post infection (dpi), and four were euthanised on day 4-dpi to analyse blood and liver RNA concentrations for Py and MA-EBOV [49].

The second laboratory study used wild-type, interferon (IFN-αβ) receptor knock-out mice (Ifnar^-I-^), and IFN-γ receptor null mice (Ifngr^-I-^) inoculated intraperitoneally with a higher dose of 10^6^ parasitized red blood cells of Py and challenged with MA-EBOV or recombinant vesicular stomatitis virus (rVSV)/EBOV glycoprotein (GP) at three different concentrations: 1-, 10- and 100-iu on the sixth day following Py infection. In each group, seven mice were followed for survival up to 18 days and three were euthanised on day 3 to determine viremia and viral load. Knock-out mice were used to study the signalling pathways implicated in initial lab findings. The study also analysed Ifnar^-I-^ mice with *P*. *chabaudi* (Pcc) infection in the first round of experiments but found no difference in outcomes compared to Py so was discontinued. To test the durability of *in vivo* effects, parallel experiments challenged Py-infected mice with rVSV/EBOV GP at weeks 1, 3, 5, and 7 post-Py inoculation. In addition, an *ex vivo* model of infection was analysed using naïve human and mouse macrophages challenged with sera from parasitaemic rodents and from rhesus macaques infected with *Plasmodium cynomolgi* [50].

### Review of key findings

#### Prevalence and seroprevalence of EBOV/*Plasmodium* spp. coinfection

Thirteen cohort studies reported data on prevalence of coinfection. Prevalence of *Plasmodium* coinfection among EVD patients was found to be 33.3% among patients with BDBV in DRC though this was only based on nine malaria RDTs. Among patients infected with ZEBOV, prevalence of *Plasmodium* coinfection in EVD(+) cohorts ranged from 18.9% to 72.2% for studies utilising malaria RDTs, and 15.4% and 19.4% for the two studies utilising qRT-PCR. In addition to these cohorts, RNA sequencing data from EBOV(+) cases in Guinea, found that 84% (156/186) contained reads mapping to Pf, higher than malaria prevalence found by RDT on the same sample of patients (33%, 40/121). However, read depth, a proxy for parasitaemia density, varied widely from 59 to 358820 FPKM (a measure for read depth) and the discrepancy between diagnostic methods reduced as number of reads mapping to *Plasmodium* increased. RNA sequencing in Boende, DRC, found 24.3% of EBOV(+) cases were coinfected with Pf. One study which focused only on children aged <18years found prevalence in this group to be 40%. Prevalence of *Plasmodium* infection was reported as higher in children aged and less common in EBOV(+) compared to EBOV(-) individuals [34, 46].

The cross-sectional survey found evidence of an epidemiological overlap of the two infections with 10.2% (n = 425) of adults in rural Guinea positive for both current *Plasmodium* infection and ZEBOV-specific IgG antibodies, a significant overabundance than would be expected by chance (χ^2^ = 59.4, df = 1, p<0.0001).

**Fig 2:**
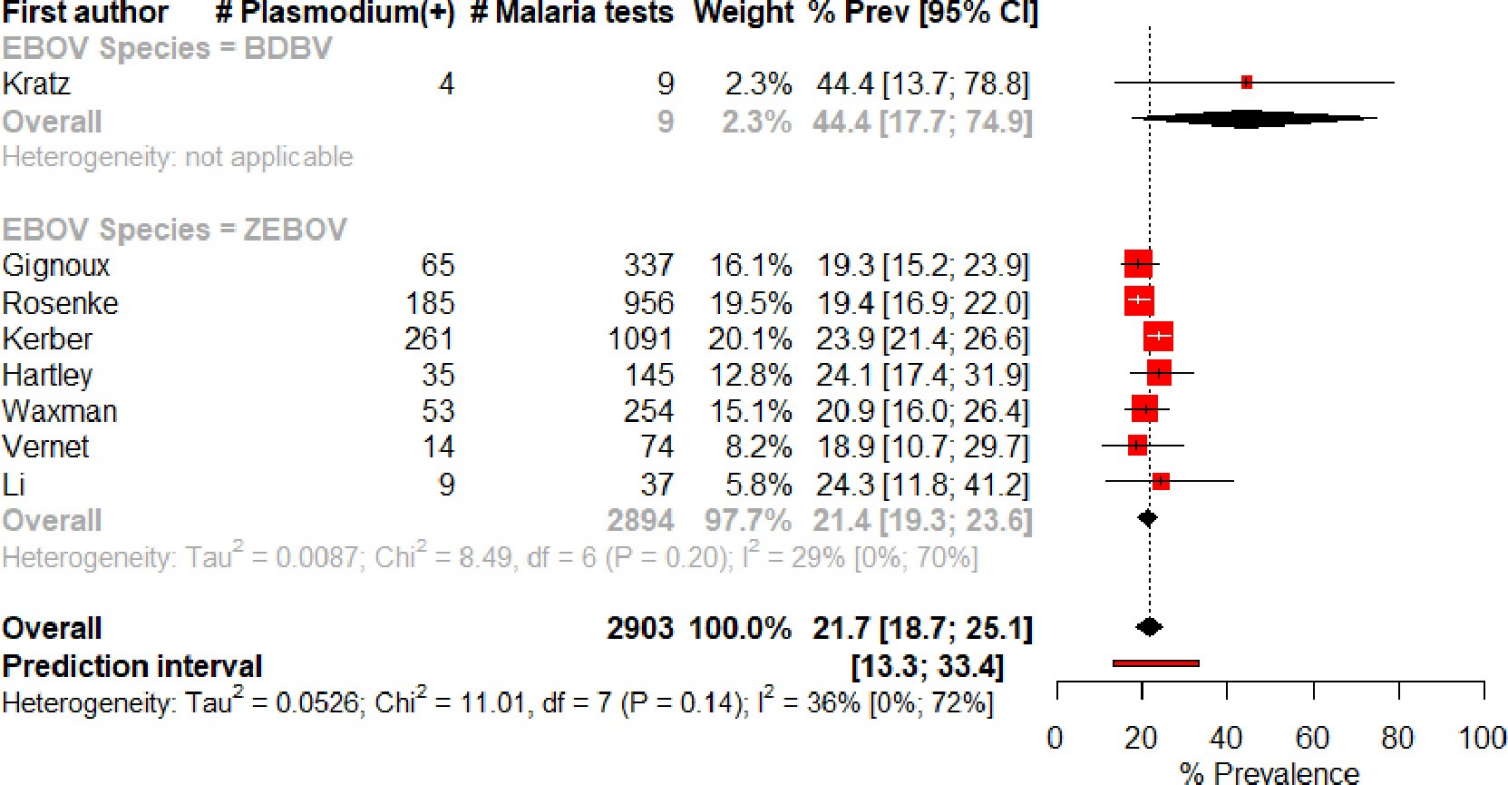
Meta-analysis of the prevalence of Plasmodium infection among EVD(+) cases, overall and split by species of Ebolavirus.

Eight observational cohorts were included in the meta-analysis. The prevalence of *Plasmodium* coinfection among EVD patients varied from 18.9% to 44.4% between studies, with the overall meta-analysis estimate calculated at 21.7%, 95% CI 18.7–25.1% (Fig 2). Between-study heterogeneity was low-to-moderate with I^2^ = 36%, 95% CI 0–72%; chi^2^ = 11.01, p<0.14; and Tau^2^ = 0.05. The prediction interval was 13.3–33.4%. Sub-group analysis including only studies with ZEBOV as the causal agent had a similar overall estimate and similar heterogeneity. Sub-group analysis by malaria diagnostic showed a similar overall estimate and high heterogeneity from studies utilising only RDTs compared to qRT-PCR or NGS an RT-PCR, though these latter methods only included one study each (S1 and S2 Figs).

#### Effect on disease pathology and the immune response

*Human subjects*. Description of the impact of *Plasmodium* coinfection on EVD pathology and immune response was given in the clinical case study and in both metagenomic studies. The clinical case report presented evidence of a potential pathophysiological interaction of the two infectious agents in the placenta [51]. Although she recovered from infection, one pregnant woman infected with Sudan virus in Uganda had a spontaneous still birth during her illness. Subsequent histological and immunohistochemical analysis showed colocalization of malarial parasite pigment and EBOV antigen within the placenta. Malarial parasite pigment was observed in fibrin and within macrophages embedded within the fibrin. EBOV antigen was similarly primarily localised to areas of fibrin deposition and maternal mononuclear cells, including the malarial parasite-pigment-laden macrophages. This interaction was not observed in a second pregnant woman infected with BDBV in DRC despite testing positive for malaria by RDT. She was, however, kept on a course of artemether-lumefantrine following the positive RDT result. Information on malaria diagnosis and treatment was not reported from the clinical case in Uganda.

In their metagenomic sequencing study, Li et al. found no significant differences in clinical characteristics (symptoms including, for example, fever, fatigue, bleeding and pain) between EVD(+)/Pf(+) and EVD(+)/Pf(-) patients [43]. Differences were observed by Carroll et al.’s comparison of biomarkers of the immune response between EBOV(+)/Pf(+) and EBOV(+)/Pf(-) patients, albeit only a small sample of each (n = 10 and 13, respectively) [39]. The abundance of transcripts of genes related to the innate response to severe malaria infection (IFIT2, IFIT3, IFITM3, ISG15, STAT1, MX1, TGF-β, PRF1, PGRMC1, CTSW, ICAM-1, CD36, IFN-γ, interleukin-10, and IFN regulatory factor 9) were not significantly different between the two groups, suggesting *Plasmodium* infection did not have any additional effect on the immune response to EBOV infection. However, biomarkers associated with the coagulation pathway (FGA, FGB, FGG, FGL1, and ALB) were increased in abundance in EBOV(+)/Pf(-) cases, suggesting, vice versa, that Pf coinfection had an inhibitory effect on coagulation.

*Animal models*. One experimental lab study measured immune response biomarkers in infected mice and found that acute *Plasmodium* infection protected against a low but lethal dose (1-iu) of virus (both MA-EBOV and rVSV/EBOV) by eliciting the proinflammatory interferon gamma (IFN-γ) pathway, rendering cells resistant to EBOV infection [50]. Morbidity and mortality were reduced, as well as viremia and viral load in the liver, spleen and kidneys of coinfected mice. The same protection was not conferred by administration of a single large bolus of recombinant IFN-γ, suggesting a persistent, subpatent *Plasmodium* infection and/or a sustained level of IFN-γ confers protection against EBOV viral replication (effect on mortality discussed below).

#### Effect on mortality

*Human subjects—CFR*. Crude EVD-related mortality was reported in 12 cohort studies and ranged from 44% (n = 39/90, ZEBOV infection, Guinea) to 72.8% (n = 118/162, ZEBOV infection, Guinea). For BDBV infection, crude CFR was 53.8% (n = 28/52, DRC) and in the study looking only at children aged <18 years crude CFR was 56.6% (n = 69/122, Liberia & Sierra Leone). CFR by *Plasmodium* infection status were reported in eleven cohorts and ranged from 42.2% to 85.7% for EBOV(+)/Pl(+) cases, and from 52.2% to 60.3% for EBOV(+)/Pl.(-) cases.

**Fig 3:**
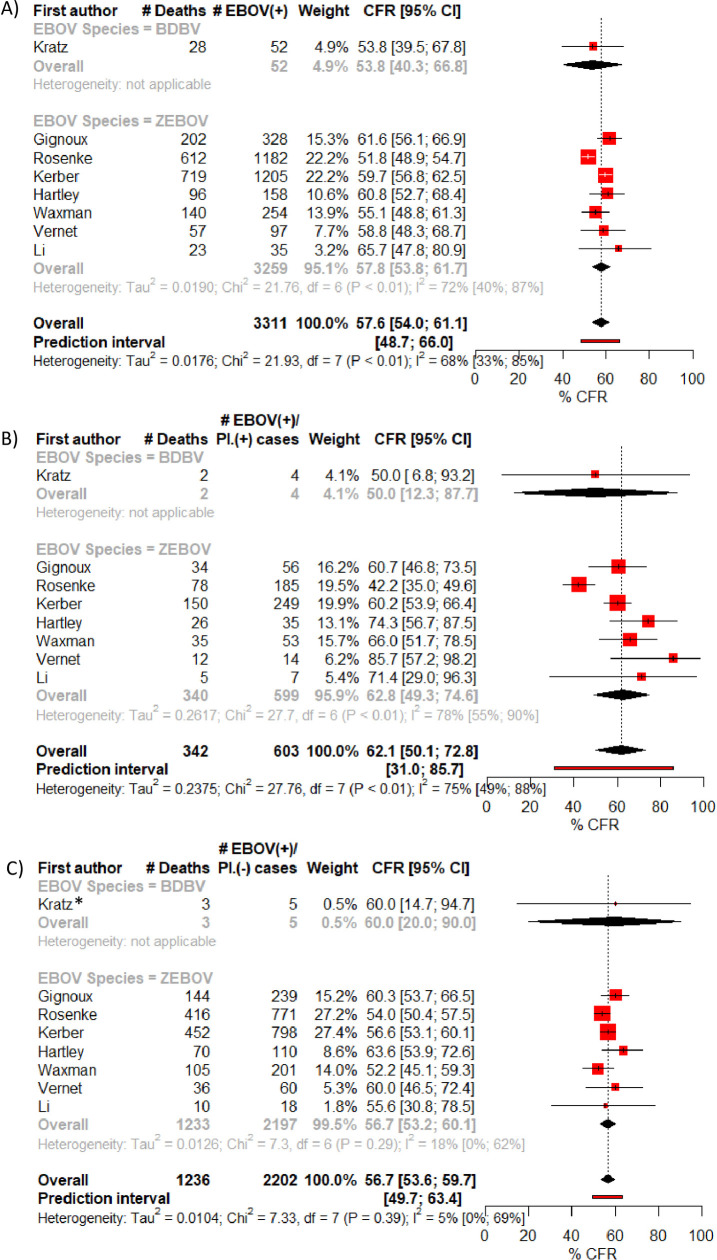
Meta-analysis of CFR: A) crude CFR, B) CFR of EBOV(+)/Pl/(+) cases, C) CFR of EBOV(+)/Pl.(-) cases.

Eight of the cohort studies were included in a meta-analysis for CFR estimates (Fig 3). Overall crude CFR was found to be 57.6%, 95% 54.0–61.1% and between-study heterogeneity was moderate-to-high with I^2^ at 68%, 95% CI 33–85%, Chi^2^ = 21931, p<0.01 and Tau^2^ = 0.02. The prediction interval was 48.7–66.0%.

CFRs by malaria infection status were estimated at 62.1%, 95% CI 50.1–72.8% for EBOV(+)/Pl.(+) cases and 56.7%, 95% CI 53.6–59.7% for EBOV(+)/Pl(-) cases (Fig 3). Heterogeneity between studies was high for the measure of CFR among coinfected cases (I^2^ = 75%, 95% CI 49–88%, Chi^2^ = 27.76, p<0.01, Tau^2^ = 0.2) but much lower for the CFR of singly-infected cases (I^2^ = 5%, 95% CI 0–69%, Chi^2^ = 7.33, p = 0.39, Tau^2^ = 0.01).

Each CFR was similar when looking only at ZEBOV species alone (Fig 3) and when looking at studies that used malaria RDTs only (S1 and S2 Figs). Although heterogeneity between study estimates of CFR among coinfected cases was reduced when analysing only studies that used malaria RDTs (S1 and S2 Figs).

*Human subjects—effect estimates*. Seven studies found no significant difference in CFR by *Plasmodium* infection status in unadjusted analyses [36, 39, 40, 42, 43, 45, 47]. Within these, Kratz et al., Vernet et al and Li et al. had a small sample sizes of coinfected individuals (n = 3, 14 and 7, respectively) and Garbern et al. had a high amount of missing malaria diagnostic data. Carroll et al. reported that mortality appeared to be increased by Plasmodium coinfection using RDT data, but this was not found using prevalence as determined by genomic sequencing data. They did find that higher burden of Plasmodium infection appeared to have higher mortality, but no absolute correlation was observed.

Two studies found no effect of coinfected in adjusted analyses. Hartley et al. found coinfection was associated with significantly higher mortality when adjusted only for age and sex (53.6% v 74.3%, OR = 3.9, p = 0.03), however, these individuals also had higher viral loads and once this was taken into account the detrimental effect was abrogated. Gignoux et al. found no effect after adjusting for multiple confounders including antimalarial prescription, age group, sex, Ct value at admission, days from symptom onset to admission, receipt of IV fluids, and number of inpatients at the Ebola treatment centre on day of admission (aRR = 1.23, 95% CI 0.52–2.86). However, this study did find that *Plasmodium* coinfection inhibited the apparent beneficial effect of ASAQ treatment; ASAQ treatment appeared to reduce mortality in EBOV(+)/Pl(-) cases compared to those treated with A-L whereas no beneficial effect was seen in EBOV(+)/Pl(+) cases.

Two studies reported increased mortality in either the general study population (Waxman et al.) or in association with age (Kerber et al.) In the former, although no significant effect was found from a smaller study drawn from the same source population (Garbern et al.), with a larger sample Waxman et al., found a significant detrimental effect of *Plasmodium* coinfection on mortality with an elevated CFR (52% v 66%) and 28-day mortality (8.2 per 1,00 person-days). In a focused subgroup analysis, adjusted for age only, there was greater mortality among EBOV(+)/Pl(+) compared to EBOV(+)/Pl.(-) cases (aHR 1.69, 95% CI 1.14–2.52, p = 0.009). Compared to EBOV(-)/Pl(-) patients, the hazard of dying was highest in EBOV(+)/Pl.(+) cases (aHR 9.4, 95% CI 6.2–14.2, p < .0001) followed by EBOV(+)/Pl.(-) cases (aHR 6.0, 95% CI 4.4–8.0, p < .0001). Kerber et al, meanwhile, found that *Plasmodium* coinfection only had an impact on mortality among 5–15-year-olds among whom it increased CFR by >20% and increased odds of death to 4.2 times greater than individuals of the same age group without *Plasmodium* co-infection. No other age groups appeared to be affected, suggesting an interaction between age and infection status.

In contrast, just one study found coinfected patients had significantly lower CFR than singly infected cases (p = 0.007) [38]. *Plasmodium* infection was associated with an increased survival when adjusted for age, sex and EBOV viral load (aRR = 1.2, 95% CI 1.1–1.4, p = 0.004). However, the effect was more pronounced when stratified by plasmodium parasitaemia level, using Ct as a proxy. High *Plasmodium* burden was then associated with increased risk of survival to 1.4, 95% CI 1.2–1.7, p<0.0001) whereas low parasite burden infection did not have a significant effect (RR 1.2, 95% CI 0.98–1.4, p = 0.08) P = 0.007). These effect estimates did not include individuals aged <5 years due to low numbers of cases in that age group.

In addition to *Plasmodium* infection, studies also reported other significant variables affecting CFR as including age, viral load, aspects of clinical treatment (e.g. referral time, length of stay) and certain clinical features (e.g. bleeding and other symptoms).

**Fig 4:**
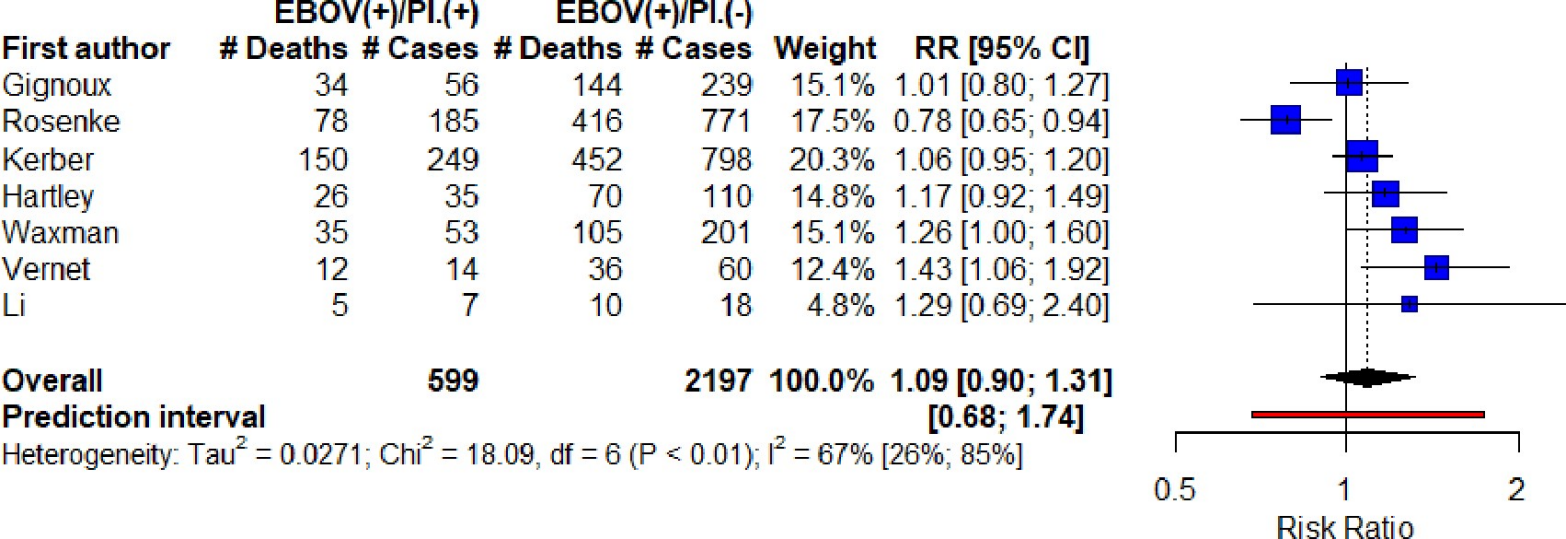
Meta-analysis of unadjusted risk ratio estimates for the effect of Plasmodium infection on EVD-related mortality (all ZEBOV).

For the meta-analysis, numbers of exposed/unexposed and with outcome data was extracted from seven cohort studies, all investigating ZEBOV (Fig 4). Individual (unadjusted) RRs ranged from 0.78, 95% CI 0.65–0.94, to 1.43, 95% CI 1.06–1.92. The overall unadjusted RR was estimated at 1.09, 95% CI 0.90–1.31. There was moderate-to-high heterogeneity (I^2^ = 67%, 95% CI 26–85%, Chi^2^ = 18.09, p<0.01, Tau^2^ = 0.03) and a prediction interval of 0.68–1.74.

**Fig 5:**
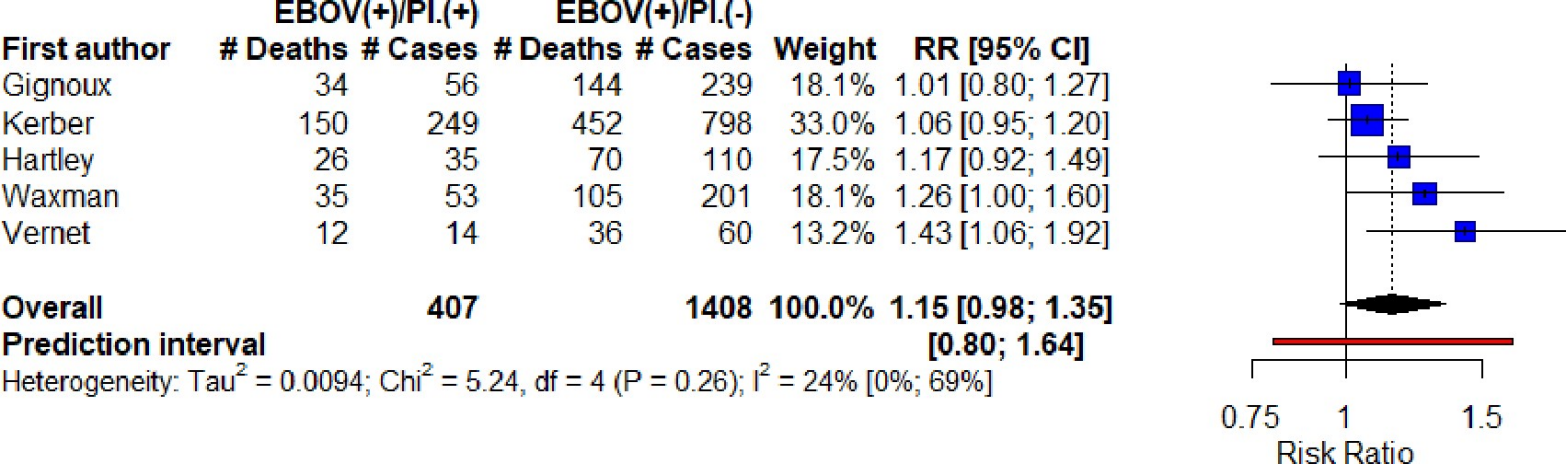
Meta-analysis of unadjusted risk ratio estimates for the effect of Plasmodium infection on EVD-related mortality (all ZEBOV) including only studies with malaria RDT as the diagnostic.

Since Rosenke et al. and Li et al. used different and more sensitive diagnostic methods for malaria detection (qRT-PCR and mNGS), these were excluded and the meta-analysis re-run only on the studies using RDT. This estimated a higher but still insignificant risk ratio of 1.15, 95% CI 0.98–1.35, with lower heterogeneity (I^2^ = 24%, 95% CI 0–69%, Chi^2^ = 5.24, p = 0.26, Tau^2^ = 0.009) and slightly reduced prediction interval of 0.80–1.64 (Fig 5).

**Fig 6:**
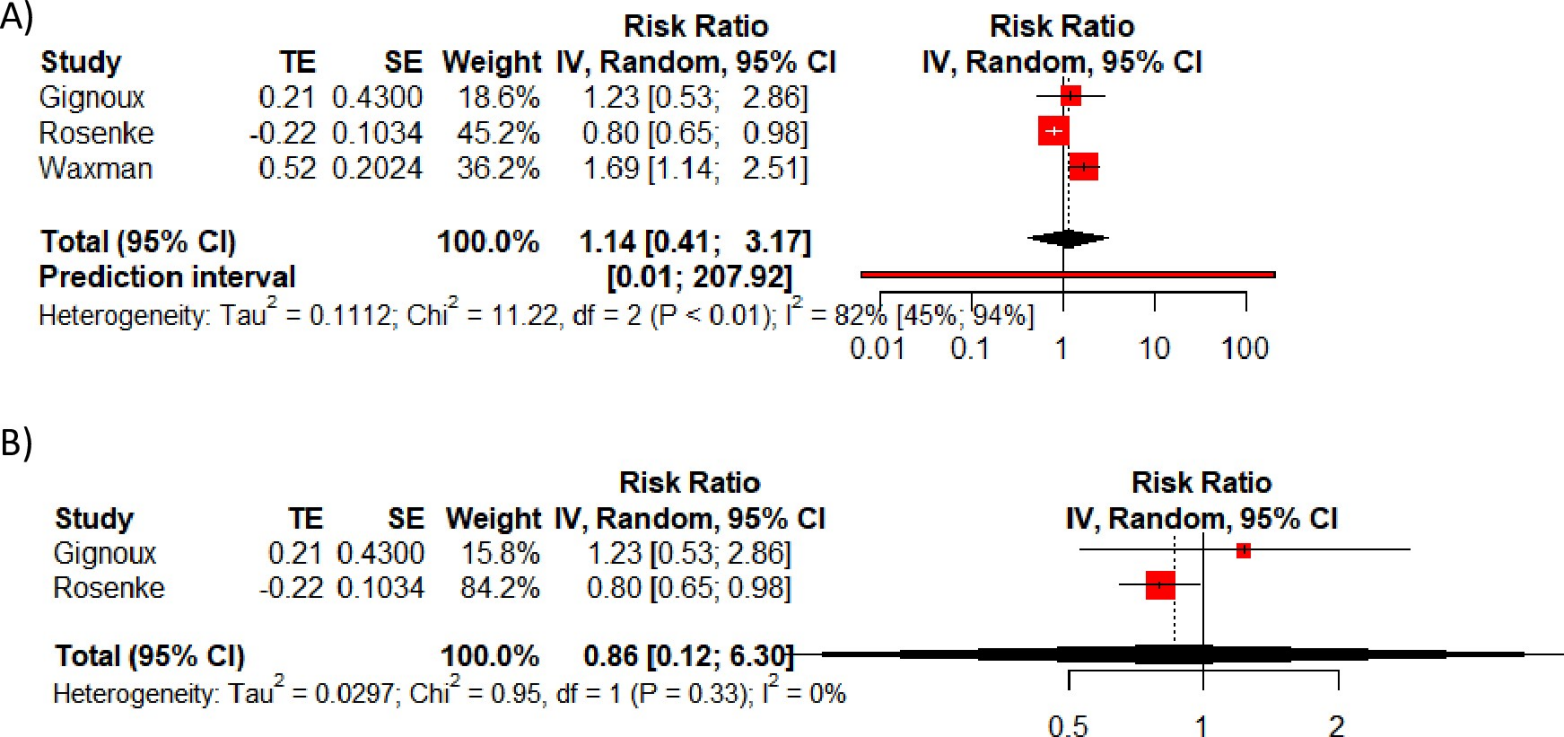
Meta-analysis of adjusted risk ratio estimates. A) all adjusted RR estimates, and B) only those that included adjustment for viral load.

Only three studies reported final adjusted effect estimates for the whole study population, though with varying confounders adjusted for. These three estimates pooled to a combined aRR estimate of 1.14, 95% CI 0.41–3.17 with high heterogeneity (I^2^ = 82%, 95% CI 45–94%, Chi^2^ = 11.22, p<0.01)) and an incomprehensibly wide prediction interval (Fig 6A). Since only two of these adjusted for EBOV viral load, which was considered to be a significant factor in predicting outcome in seven studies, a meta-analysis was conducted solely on these two studies, estimating a RR of 0.86, 95% CI 0.12–6.30 (Fig 6B).

*Animal models*. In addition to these observational studies in the field, the two laboratory studies also report the effect of coinfection on mortality in murine models. Rosenke et al. 2018 found no effect of coinfection on mortality in mice when challenged with a high dose of MA-EBOV at 100-median lethal dose. In contrast, Rogers et al. found that acute *Plasmodium* infection protected from lethal virus challenge at low (1-iu) MA_EBOV challenge but less so with moderate to high (10-iu and 100-iu) doses. This protective effect waned over time, with Py-infected mice protected from EBOV challenge three weeks after Py infection, but not by 5 weeks. This was IFN-γ dependent as discussed above.

#### Quality of studies included in meta-analysis

The eight studies included in the meta-analysis were analysed for quality using the Newcastle-Ottawa Scale (Table 2). Four studies (Kratz, Waxman, Vernet and Li) were deemed to be ‘Poor’ because, while they scored well under the selection and outcome categories, they did not score under comparability, i.e. they did not attempt to adjust analyses to take into account important confounding factors. Although the other four were deemed ‘Good’, all studies lost points on the representativeness of the exposed cohort since all only had diagnostic data from hospital-based cohorts, and four studies had follow-up rates that were deemed high enough to introduce potential bias into their final estimates (Kratz, Gignoux, Rosenke and Vernet).

**Table 2:** Outcome of Newcastle-Ottawa Scale analysis on studies included in the meta-analysis.

	Selection				Comparability	Outcome			Quality Score (/9)
Study	Representativeness of Exposed Cohort	Selection of the Non-Exposed Cohort from Same Source as Exposed Cohort	Ascertainment of Exposure	Outcome of interest not present at start of study	Comparability of cohorts based on factors controlled for	Assessment of outcome	Follow-up was long enough for outcome to occur	Adequacy of follow-up	
Kratz	Although the paper looked at community cases, malaria diagnostic data was only collected among cases that made it to the ETC which the paper reports as having different characteristics to the community cases	Yes ✵	Secure clinical data collected by trained medical staff ✵	Yes^#^ **✵**	CFR by malaria status were reported but no attempt to control for confounding factors	Record linkage **✵**	Yes—cases followed until death or discharge from ETU **✵**	Cases all followed for outcome but only 50% of ETC patients had malaria diagnostic test data available for reasons unknown.	5—Poor
Gignoux	ETC-based cohort in Foya, Liberia. Could be a biased subsample of community cases but no comparison of sample demographics to general population	Yes **✵**	Secure clinical data collected by trained medical staff **✵**	Yes^#^ **✵**	CFR/Effect measures adjusted for Ebola viral load. **✵** Other factors adjusted for included age, sex (among others) but didn’t measure malarial parasite load.	Record linkage **✵**	Yes—cases followed until death or discharge from ETU **✵**	Malaria diagnostic data missing for 44/381 EBOV(+) cases (11.5%). Outcome data missing for 53/381 EBOV(+) cases (13.9%).	6—Good
Rosenke	ETU-based cohort j Monrovia, Liberia. Could be biased subsample of community cases but no comparison of sample demographics to general population	Yes **✵**	Secure clinical data collected by trained medical staff **✵**	Yes^#^ **✵**	Outcome measures adjusted for Ebola viral load. **✵** Outcome measures also stratified by malarial parasite load and adjusted for age and sex. **✵**	Record linkage **✵**	Yes—cases followed until death or discharge from ETU **✵**	Malaria diagnostic data missing for 226/1182 EBOV(+) cases (19.1%).	7—Good
Kerber	Study looked at community deaths as well as ETU-based cases but only ETU cases had malaria diagnostic data available, thus could represent a biased sub-sample	Yes **✵**	Secure clinical data collected by trained medical staff**✵**	Yes^#^ **✵**	Outcome measures adjusted for Ebola viral load, but only viral load. **✵**	Record linkage **✵**	Yes—cases followed until death or discharge from ETU **✵**	Only 2% (26/1231) EBOV(+) patients without outcome data; unlikely to introduce significant bias. **✵**	7—Good
Hartley	ETC-based cohort in Sierra Leone. Could represent a biased sub-sample of all community cases if inequitable accessibility/uptake in the community; no comparison made of sample demographics to general population	Yes **✵**	Secure clinical data collected by trained medical staff **✵**	Yes^#^ **✵**	Outcome measures adjusted for Ebola viral load, but only viral load.**✵**	Independent blind assessment **✵**	Yes—cases followed until death or discharge from ETU **✵**	Only 13/158 EBOV(+) cases missing malaria diagnostic data (8.2%); unlikely to introduce significant bias. **✵**	7—Good
Waxman	ETU-based cohort spread over 3 ETUs. Could represent biased sub-sample of all community cases; no comparison made of sample demographics to general population or deaths in the community.	Yes **✵**	Secure clinical data collected by trained medical staff **✵**	Yes^#^ **✵**	Outcome measures adjusted for age only.	Record linkage **✵**	Yes—cases followed until death or discharge from ETU **✵**	Complete **✵**	6—Poor
Vernet	ETC-based cohort in Macenta, Guinea. Could represent biased sub-sample of all community cases; no comparison made of sample demographics to general population or deaths in the community.	Yes **✵**	Secure clinical data collected by trained medical staff **✵**	Yes^#^ **✵**	Outcome measures not adjusted for any confounding variable	Record linkage **✵**	Yes—cases followed until death or discharge from ETU **✵**	12.5% non-fatal and 26.3% fatal EVD cases without clinical follow-up, and 24% cases without data on viral titre or malaria diagnosis.	5—Poor
Li	ETU-based cohort in Boende, DRC. Could represent biased sub-sample of all community cases; no comparison made of sample demographics to general population or deaths in the community.	Yes **✵**	Secure clinical data collected by trained medical staff **✵**	Yes^#^ **✵**	Outcome measures not adjusted for any confounding variable	Record linkage **✵**	Yes—cases followed until death or discharge from ETU **✵**	5/70 patients without clinical data (7%); unlikely to introduce significant effect on results. **✵**	6—Poor

^#^Given the acute nature of Ebola outbreaks, all studies were deemed to not have the outcome of interest prior to the study start.

Comparability stars awarded if study adjusted for Ebola viral load (one star) and/or adjusted for age, sex and Plasmodium parasiteamia (one star).

Assessment of quality scale =

Good quality: 3 or 4 stars in selection domain AND 1 or 2 stars in comparability domain AND 2or 3 stars in outcome/exposure domain.

Fair quality: 2 stars in selection domain AND 1 or 2 stars in comparability domain AND 2 or 3stars in outcome/exposure domain.

Poor quality: 0 or 1 star in selection domain OR 0 stars in comparability domain OR 0 or 1 stars in outcome/exposure domain.

## Discussion

We have attempted to consolidate findings on the prevalence and effect of *Plasmodium* coinfection on mortality from EVD which could have significant implications for future outbreaks of EVD and other viral outbreaks with similar pathology. The number of studies identified, particularly those for the meta-analysis, was limited. This could be because EVD outbreaks have historically been small and, indeed, the majority of studies stem from the 2014 West Africa EVD outbreak which had a huge affected population in comparison and a high level of support from national and international NGOs and research institutions [6, 52]. EVD outbreaks also develop quickly and in countries with limited surveillance and weak health systems, limiting resources and planning for research purposes. Although we identified 13 observational studies, there were overlapping study populations meaning only eight unique studies could be included in the meta-analyses. This is largely because research data came from ETUs which were set-up to specifically manage EVD patients during the 2014 outbreak.

Overall, one-quarter of EVD cases were infected with *Plasmodium* (26.9%, 95% CI 16.8–40.0%), and, aside from one, appeared relatively consistent across individual studies. Plasmodium spp. prevalence was lower in EVD confirmed cases compared to patients without EBOV infection, most likely because presenting symptoms were due to the viral infection and not malaria in the former group. Indeed, Hartley et al. found that malaria infection could be used as one variable (among others) to help distinguish EVD cases from other diagnoses [35]. Despite this, one-fifth of EVD cases is a high proportion and would have important public health implications if coinfection were to alter morbidity or mortality in a significant way. There was a lack of consensus, however, between studies in relation to coinfection and its effect on EVD outcomes.

From the cohort studies reviewed, the majority found no significant effect of *Plasmodium* infection on mortality, while two found a significant detrimental effect, and one found a significant protective effect. Overall, the meta-analysis estimated that the CFR of coinfected patients was not significantly different from the CFR of EBOV(+)/Pl(-) patients, and a meta-analysis of effect sizes found no significant effect of coinfection on mortality (RR = 1.09, 95% CI 0.90–1.31). Although tests of heterogeneity have low statistical power with a small number of studies, use of the chi-square p-value and I^2^ confidence intervals can provide more confidence in these estimates and in this case suggest high heterogeneity between studies for both the overall effect estimate and the CFR of coinfected patients (EBOV(+)/Pl(+)) [53]. Conversely, between-study heterogeneity appeared to be lower between estimates of the CFRs of individuals infected with EBOV alone (EBOV(+)/Pl(-)). The observed heterogeneity in the estimates related to coinfection could suggest other factors complicate the outcome that have not been accounted for.

In terms of study protocols, only two studies included in the meta-analysis used a malaria diagnostic that was more sensitive than RDT: qRT-PCR and a combination of RT-PCR and mNGS. One of these [38] (using RT-PCR) was also the only one to find a positive effect of coinfection on surviving EVD. When these studies were removed from the meta-analysis, heterogeneity was reduced and the overall estimate, although it remained insignificant, was biased toward a detrimental effect of *Plasmodium* on EVD mortality. However, the difference in testing procedure doesn’t logically explain why Rosenke et al. would observe a protective effect from *Plasmodium* since RT-PCR is more sensitive than RDT, particularly in detecting low parasite density infection, and so could be expected to identify more malaria cases and would actually bias the effect estimate toward a detrimental effect (a higher RR if considering mortality as the outcome) [54, 55]. Conversely, the studies using RDTs could be biased toward a detrimental effect since they used RDTs based on HPR2 for Pf identification which has been shown to be associated with false positive results [56, 57].

Additionally, the RT-PCR and mNGS tests could detect different stages of malaria infection to the RDTs, i.e. detecting sub-patent infection compared to more active/acute infection. These stages of malaria are associated with different immune responses which could affect EVD outcomes differently. Younger age groups are more at risk of acute *Plasmodium* infection compared to adults who gain some level of immunity to severe infection and are more likely to have a chronic, subpatent infection [58, 59]. In the cohort studies presented here, younger people were found to be more likely to be associated with both coinfection and greater EVD-related CFR yet age was not adjusted for in most analyses. If a study had a younger age profile this could result in an inflated CFR among coinfected cases. Furthermore, Rosenke et al.’s analysis did not include cases age <5 years, while other evidence pointed to a potential interaction between age and Plasmodium infection status; Kerber et al. found that coinfection among 5-14-year olds increased their risk of death but the same was not seen in other age groups [46]. This could be suggestive of differences in the intensities of infection and/or immune responses between age groups in either EBOV or *Plasmodium* infection.

Acute *Plasmodium* infection is associated with activation of pro-inflammatory pathways and has been implicated in influencing response to other concurrent infectious agents such as respiratory viruses, enteric bacteria and HIV [17–20]. HIV, like EBOV, is an RNA virus, however, it is a chronic infection as opposed to the acute infection seen in EVD which could lead to alternative interactions. However, prior *Plasmodium* infection was found to reduce viremia and associated pathologies of chikungunya virus, a more acute RNA virus, via proinflammatory responses through IFN-γ production [60]. Despite this, only one study in our meta-analysis attempted to discern the effect of more acute *Plasmodium* infection. Rosenke et al. looked at parasite burden (using Ct as a proxy) and found that higher parasite load (Ct ≤ 20), which would be expected from more acute infection, was associated with improved survival (reduced mortality) compared to those with a lower parasite burden for which protection was not significant. A similar protective effect from acute infection was seen in the laboratory study by Rogers et al which found acute *Plasmodium* infection protected mice and human macrophages from EBOV challenge via a low but sustained IFN-γ response which protected from EBOV challenge up to three weeks post *Plasmodium*-infection but dwindled thereafter. However, in the only study to monitor similar human biomarkers, Carroll et al found that markers of the innate immune response to acute *Plasmodium* infection, including IFN- γ, were not different between Pf(-) and Pf(+) EVD cases whereas biomarkers associated with the coagulation pathway were reduced in patients Pf(+). This analysis thus suggests the inflammatory pathway found to be important in mice is perhaps not important in human cases, although, the analysis by Carroll et al was based on a very small sample size of individuals (10 and 13 per group).

The protective effect observed from the laboratory study by Rogers et al. was not observed in the second laboratory study identified, however, this second study used only a very high lethal dose of MA-EBOV (100-iu) [49]. Use of similarly high doses by Rogers et al. (10-iu and 100-iu) also showed limited effect on morbidity and outcomes compared to the low EBOV dose (1-iu) which was significantly protective, thus suggesting high viral load overrides any potential protection from coinfection [50]. In human studies, viral load was only quantified in seven studies and, where analysed, all found that higher viral load led to greater mortality. Despite this, only three studies reported analyses that adjusted for viral load. Among these, Hartley et al. found that coinfected cases had a higher viral load than EBOV-only infected cases and when accounted for in their analyses viral load abrogated the apparent detrimental effect of *Plasmodium* infection on mortality [34]. The two other studies that adjusted for viral load included the positive effect estimate from Rosenke et al. and an insignificant finding from Gignoux et al. [36, 38]. Not accounting for this confounding variable is a clear limitation in the other effect estimates reported.

Due to the failure to adjust for important confounders, four of the eight studies included in the meta-analysis were deemed poor quality based on the Newcastle-Ottawa Scale analysis. As well as not adjusting for viral or parasite load, many of the studies failed to account for other potential confounders including age, sex, and time from symptom onset to admission. The majority of studies did not specify *Plasmodium* species and it could be expected that different species would be associated with a different proportion of chronic versus acute infections and differences in immune response. However, it is unlikely to explain differences in this context since the vast majority of malaria infection is caused by Pf in each of these countries [61]. Similarly, it is conceivable that different EBOV species could have different immune responses that may interact differently. However, all but one observational study examined ZEBOV and thus does not explain differences observed.

Outside of the cohort and laboratory studies, the case report of a pregnant woman in Uganda implicated other potential interactions between the two infectious agents in the placenta of pregnant women [51]. It is unclear whether the co-localization of these two pathogenic agents had an impact on the outcome of still birth experienced in this case. As a one-off observation, this does need to be explored in other future cases to see if there is a legitimate interaction of concern.

So, what does all this mean and what are the implications for future outbreaks of EVD and other viral diseases? From the literature as it stands, it remains unclear whether *Plasmodium* coinfection has an effect on EVD morbidity and mortality. Although pooled meta-analyses suggest no significant effect, there is a high amount of variability between study effect estimates that have not all had appropriate confounding variables considered, and/or had follow-up rates that may have biased results. With the advent of the new Ebola vaccine (rVSV-ZEBOV-GP, manufactured by Merck) which has shown high efficacy in trials, the clinical interaction of comorbidities with EBOV may have less significance in future EVD outbreaks [21]. However, it will take time for a vaccine to be delivered widely and is not yet fully assessed in all populations. Furthermore, the learnings described here in terms of research conducted and gaps remaining can be applied to improve our knowledge of malaria coinfections on other viral outbreaks in the future. Since other viral diseases with similar immune response profiles could be affected similarly by Plasmodium infection, improvements in study design and data collection could be made to ensure that all essential clinical information is collected during subsequent outbreaks. In this case, features of clinical presentation, details of clinical acute care, data on viral loads, parasite densities and immune system biomarkers in particular are important and lacking.

Planning for research purposes is difficult in an outbreak setting where the outbreak is fast-moving and resources are limited but being better prepared with a planned set of guidance on what data should be collected prior to the next outbreak would enable faster implementation of data collection for subsequent research. Following analyses, all studies should be encouraged to publish all results related to these important factors, including raw numbers, unadjusted and adjusted estimates, to better enable meta-analytics.

Even despite these improvements, due to the complex nature of the immune system, it may be that experimental lab methods will give the best insights. Findings by Rogers et al are interesting, but more comparison needs to be done to look into other disease models, as well as how well these animal models transfer to the human context. Mouse models of malaria and EVD do not mimic all facets of the diseases in humans, but findings from these models can be used to narrow down the field of investigation in the clinical setting—e.g. explicit measurement of immune system biomarkers such as IFN-γ, stratified by Plasmodium parasite load and viral load, should now be recommended in future clinical research.

Remaining limitations on implementing better-planned research may be sample size restrictions if future outbreaks are not large enough, and diagnostic and clinical capacities to conduct the research. In this case, retrospective genomics analysis on stored blood samples may offer a useful tool since this is increasingly affordable and samples can be stored and analysed in retrospect or in partner institutions with greater research capacity. RNA/DNA read data can provide quantifiable information on prevalence of infection, viral and parasite loads, and biomarkers in one protocol, and thus perhaps be able to answer some of gaps in the data presented here.

## Supporting information

S1 Checklist (DOC)

S1 Fig Prevalence of Plasmodium coinfection among EVD cases by malaria diagnostic. (DOCX)

S2 Fig CFR of EVD cases by Plasmodium infection including only studies using RDT for malaria diagnosis. (DOCX)
