# Supplementary material for: The impact of malaria coinfection on Ebola virus disease outcomes: A systematic review and meta-analysis
Source: PLoS One. 2021 May 24;16(5):e0251101. doi: 10.1371/journal.pone.0251101 (PMC8143409; doi:10.1371/journal.pone.0251101)

S2 Fig. CFR of EVD cases by Plasmodium infection including only studies using RDT for malaria diagnosis.


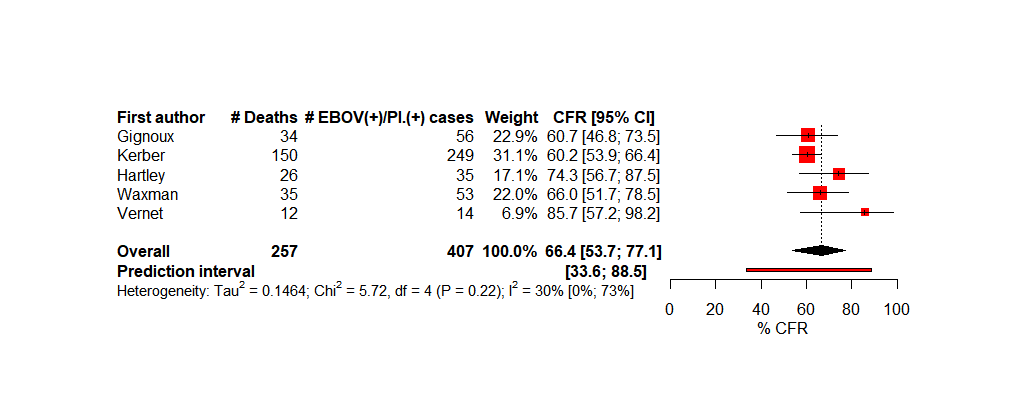

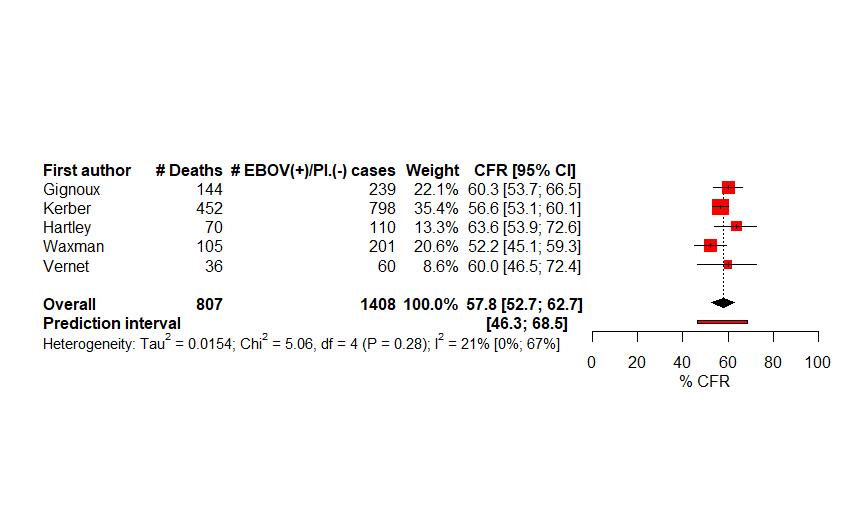

Supplement: S2 Fig — (DOCX) [file pone.0251101.s003.docx]
